# Selection into medicine: the predictive validity of an outcome-based procedure

**DOI:** 10.1186/s12909-018-1316-x

**Published:** 2018-09-17

**Authors:** Sanne Schreurs, Kitty B. Cleutjens, Arno M. M. Muijtjens, Jennifer Cleland, Mirjam G. A. oude Egbrink

**Affiliations:** 1 Department of Educational Development and Research, School of Health Professions Education, Faculty of Health, Medicine and Life Sciences, Maastricht University, Universiteitssingel 60, P.O. Box 616, 6229 ER Maastricht, the Netherlands; 2 Department of Pathology, Faculty of Health, Medicine and Life Sciences, Maastricht University, Maastricht, the Netherlands; 3 Institute of Education for Medical and Dental Sciences, University of Aberdeen, Foresterhill, Aberdeen, UK; 4 Department of Physiology, Faculty of Health, Medicine and Life Sciences, Maastricht University, Maastricht, the Netherlands

**Keywords:** Selection, Backward chaining, Outcome-based, Predictive validity

## Abstract

**Background:**

Medical schools must select students from a large pool of well-qualified applicants. A challenging issue set forward in the broader literature is that of which cognitive and (inter)personal qualities should be measured to predict diverse later performance. To address this gap, we designed a ‘backward chaining’ approach to selection, based on the competences of a ‘good doctor’. Our aim was to examine if this outcome-based selection procedure was predictive of study success in a medical bachelor program.

**Methods:**

We designed a multi-tool selection procedure, blueprinted to the CanMEDS competency framework. The relationship between performance at selection and later study success across a three-year bachelor program was examined in three cohorts. Study results were compared between selection-positive and selection-negative (i.e. primarily rejected) students.

**Results:**

Selection-positive students outperformed their selection-negative counterparts throughout the entire bachelor program on assessments measuring cognitive (e.g. written exams), (inter)personal and combined outcomes (i.e. OSCEs). Of the 30 outcome variables, selection-positive students scored significantly higher in 11 cases. Fifteen other, non-significant between-group differences were also in favor of the selection-positives. An overall comparison using a sign test indicated a significant difference between both groups (*p* < 0.001), despite equal pre-university GPAs.

**Conclusions:**

The use of an outcome-based selection approach seems to address some of the predictive validity limitations of commonly-used selection tools. Selection-positive students significantly outperformed their selection-negative counterparts across a range of cognitive, (inter)personal, and mixed outcomes throughout the entire three-year bachelor in medicine.

## Background

As there are many more applicants than places, medical schools need to select students from a large pool of suitably qualified candidates. Schools must also ensure they admit those candidates most likely to succeed and, crucially, become good doctors [1–3]. A number of important issues influence selection for admission [3, 4]. One of these is ensuring that selection tools assess the attributes considered important by key stakeholders, including patients. Traditionally, selection into medical school was solely based on prior academic attainment. Currently, there is increasing recognition that broader criteria are required, as there is more to being a capable medical student or doctor than academic performance [5–7]. Most medical schools now aim to select applicants who are both academically capable and also possess (inter)personal skills befitting a career in medicine, such as team-working and communication skills [8, 9].

Developing a selection procedure that can fairly and accurately discriminate between applicants, based on academic as well as (inter)personal criteria, is challenging [10–13]. Many schools struggle with the question of what combination of tools to use to ensure that all desirable academic and (inter)personal qualities are assessed [14]. Our observation is that, on a local level, the choice of selection tools is often rooted in tradition, resource concerns and/or essential but narrow criteria, such as psychometric qualities [1, 2, 15]. In addition, different selection tools are better at predicting different outcomes. For example, tools measuring cognitive abilities (e.g. Grade Point Average, GPA) seem better at predicting academically-loaded assessments in the earlier years of medical school [2, 16], whereas ‘(inter)personal’ assessments (e.g. Multiple Mini Interviews, MMIs, and Situational Judgement Tests, SJTs) seem better at predicting more clinically-oriented performance in the later years of medical education [1]. Cognitive and (inter)personal assessments have been integrated in some tools, but the predictive value of these integrated tools is moderate at best [1, 2, 9].

**Fig. 1:**
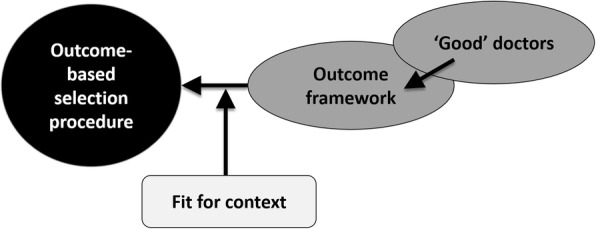
Visual representation of the use of backward chaining from the desired end goal (‘good doctors’) to create an outcome-based selection procedure

One potential way to address the aforementioned dilemmas is to develop a more holistic and outcome-based approach to selection into medical school. One way of doing this is to define the competences of a ‘good doctor’ and use these as the basis of a selection procedure [15, 17]. These competencies can be derived from outcome frameworks, which describe the competences and expertise that medical students must achieve by graduation to ensure that they have acquired the basics for being good doctors and meeting patient/healthcare needs (examples of outcome frameworks: [18–20]). Different frameworks are used worldwide, but they share analogous objectives and differ mostly in level of detail, context and terminology [12]. As a result of this commonality, ‘backward chaining’ (i.e. working backwards from the goal) from one exemplary framework into an outcome-based selection procedure will be broadly relevant across medical schools. Furthermore, the context in which the selection procedure is applied should be taken into account, e.g. undergraduate versus graduate selection, learning environment, and other contextual factors of importance to the institution (see Fig. 1). The proposed procedure is in line with recently stated developments in competency-based medical education, where it is paramount to combine multiple assessments by multiple assessors. Indeed, developing a multi-tool, outcome-based approach selection blueprinted to a framework of competencies is aligned with the global move towards competency-based approaches to preparing the next generation of health professionals [17, 21].

However, before recommending multi-tool, outcome-based selection as the way forward, it is critical to examine whether this approach does indeed predict performance across competences. Especially in current times of limited resources and increased accountability demands, it is important to employ an evidence-based selection procedure. Therefore, the aim of this study was to examine whether an outcome-based, holistic selection procedure is predictive of study success in a medical bachelor curriculum. The selection procedure as well as the curriculum and assessment program under study are aligned with the CanMEDS framework of competences [20], which is used to define the qualifications for medical doctors in the Netherlands [19]. Due to the transition from lottery to selection that occurred during the period of study (see Methods), we had the unique opportunity to compare study results of students who were selected (selection-positives) to those of students who were rejected in the same selection procedure, but still got into medical school via the national weighted lottery (selection-negatives). Therefore, our concrete research question was: how does performance in a medical bachelor curriculum differ between students that were selected (selection-positives) or rejected (selection-negatives) in the same outcome-based selection procedure?

## Methods

### Context

This study was performed at Maastricht University Medical School (MUMS). As is typical in the Netherlands, MUMS comprises a three-year bachelor and three-year master phase. We focused on the bachelor phase, which encompasses a mix of theoretical and practical educational elements.

This study included three cohorts of students, starting in 2011, 2012 and 2013. In 2011 through 2013, 50 (2011) to 60% (2012 and 2013) of the available study places was assigned through the local, outcome-based selection procedure; this limitation was imposed by the national government. Remaining places were filled via the national weighted lottery, available to applicants who were rejected in the selection procedure or who did not participate in selection at all. This unique situation enabled comparison of selection-positive students’ study outcomes with those of selection-negative (i.e. primarily rejected) students. The third group of students, who entered MUMS through lottery only (without participating in the selection procedure), was not included in the present study since their study outcomes could not be related to their performance in the selection procedure. Before 2011, all admissions into MUMS were assigned through the national weighted lottery, while from 2014 onwards MUMS transitioned to full selection of the cohorts. For more information on selection in the Netherlands, the reader is referred to Schripsema et al. [22].

### Selection procedure

The selection procedure applied in 2011–13 consisted of two stages, both based on the CanMEDS framework of competences (Table 1; [20, 23]).

**Table 1:** Translation of the CanMEDS competences into a blueprint of derived competences for the selection procedure

CanMEDS	Derived competences
Medical performance & Knowledge and science^a^	Knowledge shown at pre-university education (pu-GPA^b^) Transfer (knowledge and information integration) Textual comprehension and structuring, verbal reasoning & inductive reasoning
Communication	Overall communication skills & strength of arguments
Collaboration	Collaboration skills
Managing	Organizational skills
Health advocating	Social and medical consciousness
Professionalism	Ethical awareness Empathy Reflection skills

^a^combination of two CanMEDS competences

^b^*pu*-GPA pre-university Grade Point Average

In the *first round* applicants completed a pre-structured online portfolio, which comprised four parts. The first part (worth 40% of the total score for the portfolio) was pre-university training (including pre-university GPA; pu-GPA). The second part (also 40%) was a description of previous extracurricular activities, requesting skills relevant for a medical student and/or doctor (e.g. communication, collaboration, organization, and professionalism). The last two parts, each worth 10% of the portfolio score, concerned knowledge of and opinion on the medical curriculum and the Problem-Based Learning (PBL) system at MUMS; these parts aimed at establishing the applicants’ fit for context (Fig. 1). Applicants were ranked according to the weighted average of scores for the four parts. A predetermined number of highest ranking applicants in the first round (twice the amount of places to be allotted via selection) were invited to the second round of the selection procedure. The scores for the first-round portfolio were not taken into account in the second round.

The *second round*, a selection day at MUMS, consisted of a Video-based Situational Judgment Test (V-SJT) and a combination of aptitude tests. The derived competences based on the exemplary framework of competences (CanMEDS; Table 1) formed the blueprint for the assignments in the second round; backward chaining was used to implement these competences into the assignments. The V-SJT was based on the Computer-based Assessment for Sampling Personal characteristics (CASPer; [24, 25]), and consisted of eight to ten relevant video vignettes accompanied by questions assessing communication, collaboration, social and medical consciousness, ethical awareness, empathy, and reflection. Aptitude tests have shown to be of added value to selection procedures [1, 2, 26]. The aptitude tests used consisted of eight assignments probing talent for transfer (applying knowledge to new information), textual skills, verbal and inductive reasoning, and organization, as well as the skills assessed by the V-SJT.

For all assignments in the V-SJT and aptitude tests, predetermined answer keys were constructed by a panel of Subject Matter Experts (SMEs; [27]). In the first cohort, applicants’ answers on each assignment were assessed by two SMEs. Inter- and intra-examiner variation were consistently below 5%. Therefore, in later cohorts, all answers were assessed by a single SME per assignment; intra-examiner variation remained low each year (< 2%). The reliability of the scores (Cronbach’s alpha) was 0.71–0.76 per cohort for the V-SJT assignments and 0.54–0.58 for the aptitude tests. At the end of the selection day, candidates rated their satisfaction with the selection procedure and the extent to which the selection procedure assessed characteristics of importance for a medical career as 3.9 ± 0.9 on a scale of 1–5, in which 1 meant strongly disagree and 5 strongly agree.

To determine the final outcome of round two, Z-scores for each assignment were calculated, and applicants were ranked based on their average Z-score for all assignments. A predetermined number of the highest ranking students were admitted to MUMS (selection-positive students). Students who were rejected in either the first or second round of the selection procedure could take part in the national weighted lottery; virtually all primarily rejected students used this opportunity (> 98%). If these primarily rejected students were admitted through the lottery (selection-negative students), they entered the same curriculum as the selection-positive students.

### Outcome variables

The study outcomes available in the bachelor phase varied from cognitively-focused to mainly (inter)personal ones (Table 2). Cognitive outcomes included results obtained in theoretical tests at the end of each 4–10 week block (mean Cronbach’s α per test: 0.74–0.81), Critical Appraisal of a Topic (CAT) assignments in year 3 (Y3: [28]), and progress tests taken four times a year (mean Cronbach’s α per test: 0.64–0.76; [29]).

**Table 2:** Outcome variables based on study results obtained by students during the bachelor phase, with their possible values

	Type of assessment / outcome	Measurement level	Possible values
Cognitive	Block tests		
	Year 1&2	Continuous	Average of grades at first attempt; 0 (lowest) to 10 (highest) per year
	Year 3	Nominal	Average of grades at first attempt; F/P/G/E
	Progress test	Continuous	Mean Z-score per year, ranging from −2.3 to 4.3
	CAT^a^	Nominal	Grade at first attempt; F/P/G
(Inter) personal	CORE^b^	Nominal	End-of-year grade; F/P/G
	Portfolio year 1	Nominal	End-of-year grade; F/P
	Professional behavior		
	Year 1&2	Nominal	End-of-year grade; F/P
	Year 3	Nominal	End-of-year grade; F/P/G/E
Mixed^1^	OSCE^c^	Nominal	Once per year; F/P/G
General	Drop-out year 1	Nominal	Yes/No
	Drop-out bachelor	Nominal	Yes/No
	Study delay	Nominal	Yes/No
	ECTS^d^ after 3 years	Continuous	Amount after three years in medical school; 0–180

Mixed^1^ means that the assessment combines cognitive and (inter)personal skills

*F* Fail, *P* Pass, *G* Good, and *E* Excellent

^a^*CAT =* Critical Appraisal of a Topic

^b^*CORE =* Consultation skills and Reflection program

^c^*OSCE =* Objective Structured Clinical Examination

^d^*ECTS =* European Credit Transfer System

(Inter)personal outcomes included qualitative evaluations of the students’ consulting and reflecting skills (CORE), professional behavior, and first-year portfolio. Evaluation of CORE is based on videotaped simulated patient contacts, peer and expert feedback and self-reflection. Evaluation of professional behavior occurred throughout the whole bachelor in different settings (tutorial groups, group assignments, etc.). In the first-year portfolio, students had to reflect on their own overall performance and progression. Evaluations of these three (inter)personal aspects led to end-of-year assessments with qualifications fail, pass or good.

The OSCE, Objective Structured Clinical Examination, organized in all three bachelor years, was categorized as a ‘mixed assessment’ in which students had to apply knowledge and skills in (simulated) situations and use interpersonal skills to interact with patients. Multiple CanMEDS competences are assessed within each OSCE assessment (mean Cronbach’s α per test: 0.66–0.76).

Three general outcomes were included in the analysis: drop-out (defined as leaving MUMS without graduating), study delay (graduating from the bachelor in more than three years), and number of credit points obtained within three years (European Credit Transfer System, ECTS; 60 credits per year, accumulating to 180 credits in the three-year bachelor).

The outcome data were stored in the university’s electronic administration system, and retrieved with permission (see below) for research purposes.

### Ethical approval

During the selection procedure, applicants were asked to give their informed consent for the use of their selection and assessment data for research purposes. It was made clear that not taking part in the study would not adversely influence their progression. All selection applicants agreed to participate. Participant data was anonymized before it was shared with the research team. The study was approved by the Ethical Review Board of the Netherlands Association for Medical Education (NVMO; file number 303).

### Statistical analyses

Descriptive statistics were obtained for the demographic variables sex, age and pu-GPA, and for the outcome variables indicated above.

Exploratory Chi-Square analyses comparing the selection-positive and selection-negative students on the nominal dependent variables were conducted to obtain a first impression of the results. A repeated measures ANOVA was used to assess the overall progress test difference between groups. A sign test was conducted to investigate the overall difference between the groups taking all outcome measures into account [30].

Confirmatory multiple regression analyses were performed on student level with study performance outcomes as dependent variables, and group membership as independent variable. Group membership was represented by the binary variable groups_SP_SN (0: SN-group: selection-negative students, 1: SP-group: selection-positive students). Cohort and sex (0: male, 1: female) were considered as potential confounders and therefore included as independent variables in the model. The nominal variable cohort corresponds to three categories that are represented in the analysis by two binary (dummy) variables.

Nominal dependent variables were analyzed using logistic regression. Qualitative scores with three or more levels were dummy-coded into fail versus all other scores (i.e. *Fail/non-Fail*) and the highest possible score versus all other scores (e.g. *Good/non-Good*). Each of these binary variables was investigated as dependent variable in a logistic regression analysis with independent variables groups_SP_SN, cohort, and sex. For groups_SP_SN, the independent variable of interest, the resulting logistic regression coefficient B, Odds Ratio (OR), Wald statistic and *p*-value were reported [31]. The OR was used as an indicator of effect size, and Rosenthal’s classification values of 1.5, 2.5, and 4 (or equivalent reciprocal values 0.67, 0.40, and 0.25) to indicate small, medium, and large effects, respectively [32].

Continuous dependent variables were similarly analyzed in a linear regression analysis. For each analysis the regression coefficient b, the Standardized Regression Coefficient (SRC), and the corresponding t- and *p*-value (Student’s t-test, two-sided) of groupSR were reported. Here, the SRC was used as an indicator of effect size, using Cohen’s classification values 0.1, 0.3, and 0.5 to indicate small, medium, and large effects, respectively [33].

Analyses were conducted using the IBM SPSS Statistics 24.0 software for Windows (SPSS, Inc., Chicago, IL, USA), and results were considered statistically significant if *p* < 0.05.

## Results

**Fig. 2:**
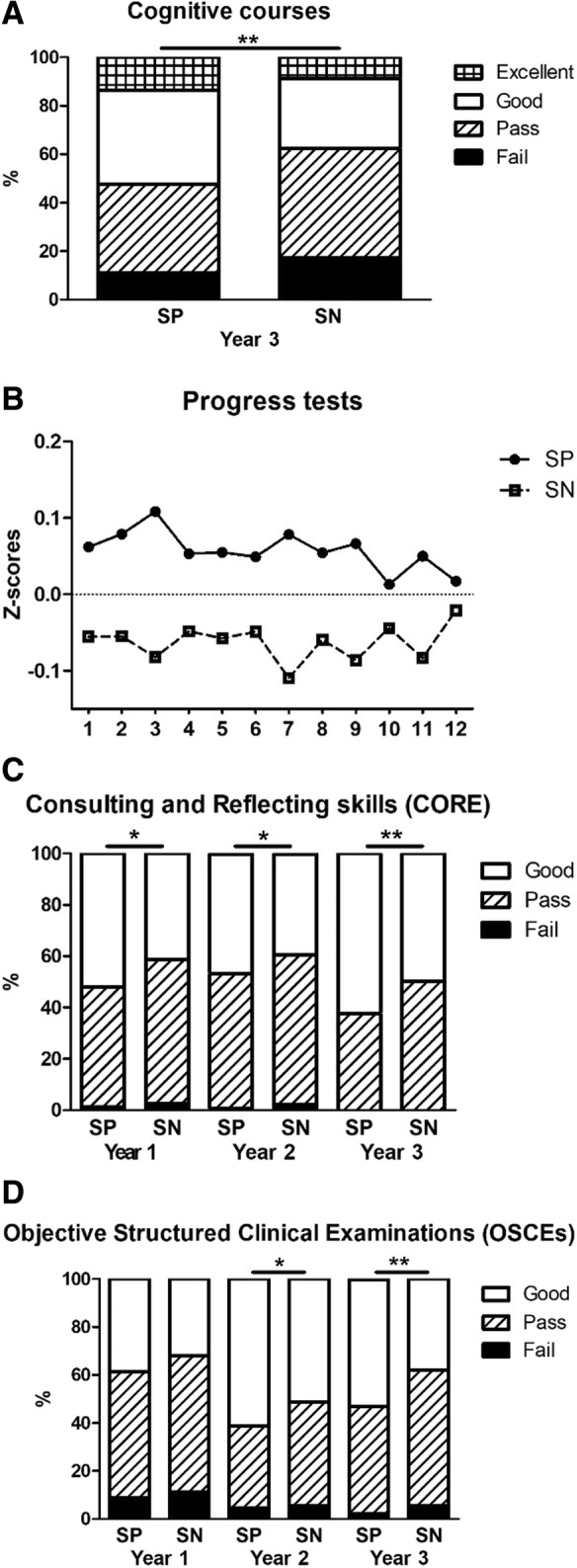
Study outcomes of selection-positive (SP) and selection-negative (SN) students on cognitive assignments, i.e. the end-of-course cognitive tests in year 3 (**a**) and the progress tests (**b**), the (inter)personally oriented CORE program (**c**) and the OSCEs (**d**) throughout the three-year bachelor phase. * *p* < 0.05; ** *p* < 0.005

Descriptive statistics, categorized by cohort and admission route (selection-positive versus selection-negative), are shown in Table 3. The combined cohorts add up to 401 selection-positive and 291 selection-negative students. An independent samples t-test confirms that these groups are significantly different in terms of their performance on the selection assessments in both rounds (*p* < 0.001). Exploratory analyses, performed to obtain a first impression of results, showed significantly better performance of selection-positive compared to selection-negative students, with respect to several cognitive, (inter)personal and mixed outcomes (Fig. 2). In the following confirmatory analyses, data from the three cohorts (2011–13) were combined while controlling for possible differences between cohort and sex.

**Table 3:** Descriptive statistics of sex, age and pu-GPA per cohort, route of admission and total

	2011 *n* = 216	2012 *n* = 238	2013 n = 238	SP-group *n* = 401	SN-group *n* = 291	Total *n* = 692
Sex (%)						
Female	63.9	68.9	71.4	70.1	65.6	68.2
Age (yr)						
Mean (SD)	19.5 (1.4)	18.8 (1.4)	19.3 (1.5)	19.2 (1.5)	19.1 (1.5)	19.2 (1.5)
Pu-GPA^a^						
Mean (SD)	6.9 (0.6)	6.9 (0.6)	6.9 (0.6)	6.9 (0.6)	6.9 (0.6)	6.9 (0.6)

*SP-group:* Selection-Positive students, *SN-group:* Selection-Negative students

^a^*pu-GPA =* pre-university Grade Point Average

### Cognitive outcomes

During the three-year bachelor program, the selection-positive students outperformed the selection-negative students on several cognitive assignments (Table 4). For the cognitive block tests, statistically significant differences were found in year 1 and 3, in favor of the selection-positive students. Furthermore, the mean progress test score was significantly higher for the selection-positive students in the first and second year of the bachelor.

### (Inter)personal outcomes

Selection-positive students scored higher than selection-negative students on (inter)personal assessments, although not all differences reached statistical significance (Table 4). The selection-positive students performed significantly better on the CORE assessments in the first and last year of the bachelor. Very few students failed professional behavior, yet, selection-positive students appear to be more likely to receive *Excellent* scores at the end of their bachelor (*p* = 0.07). Lastly, the selection-positive students scored significantly fewer fails on the first-year portfolio.

### Mixed outcomes

Notably, selection-positive students significantly outperformed selection-negative students on the OSCEs in all three bachelor years (see Table 4).

### General study outcomes

The drop-out rate in year 1 was very low and even fewer students dropped out later, without a specific difference between the groups (Table 4). The percentage of delayed students and the amount of ECTS obtained within three years did not significantly differ between the groups.

In summary, controlling for the possible confounders cohort and sex (Table 4), the selection-positive students significantly outperformed the selection-negative students on 11 of the 30 outcome variables. In addition, 15 of the remaining 19 non-significant differences were in favor of the selection-positives. These differences occurred across the whole range of variables from cognitive to (inter)personal. The effect sizes of the between-group differences, based on the ORs and SRCs, varied from small to medium/large. Of the four remaining outcome variables, two were equal for both groups; only two outcomes were found to be slightly in favor of the selection-negative students. Applying a sign test to the 30 between-group differences for all outcome variables supports the overall conclusion that study results of selection-positive students are significantly better than those of selection-negative students (*p* < 0.001).

**Table 4:** Comparison of all study performance outcome variables of selection-positive (SP) and selection-negative (SN) students. For all analyses, route of entry was coded SN = 0 and SP = 1, making SN the reference group; cohort and sex were controlled for

**Cognitive outcomes**						
Cognitive courses	SP; M (SD)^a^	SN; M (SD)	B^b^	SRC^c^	t-value	*p*-value
Year 1	7.00 (0.88)	6.85 (0.94)	0.151	0.082	2.106	0.036*
Year 2	6.82 (0.88)	6.68 (0.89)	0.106	0.059	1.520	0.129
Cognitive courses	% of SP	% of SN	B	OR^d^	Wald^e^	p-value
Year 3						
Fail/Non-fail	11.0	17.2	-0.507	0.602	4.225	0.040*
Excellent/Non-excellent	13.6	8.8	0.424	1.528	2.369	0.124
CAT^f^	SP; M (SD)^a^	SN; M (SD)	B^b^	SRC^c^	t-value	*p*-value
Year 3						
Fail/Non-fail	10.8	15.9	-0.467	0.627	3.403	0.065
Good/Non-good	5.9	9.3	-0.481	0.618	2.308	0.129
Progress tests (Z-scores)	SP; M (SD)	SN; M (SD)	B	SRC	t-value	p-value
Year 1	0.07 (0.78)	-0.06 (0.82)	0.141	0.087	2.243	0.025*
Year 2	0.06 (0.83)	-0.07 (0.85)	0.137	0.080	2.013	0.045*
Year 3	0.05 (0.85)	-0.04 (0.88)	0.090	0.052	1.256	0.210
**(Inter)personal outcomes**						
CORE^g^	% of SP	% of SN	B	OR	Wald	*p*-value
Year 1						
Fail/Non-fail	1.3	2.5	-0.546	0.579	0.830	0.362
Good/Non-good	52.1	41.4	0.464	1.591	8.068	0.005*
Year 2						
Fail/Non-fail	0.5	2.3	-1.299	0.273	2.428	0.119
Good/Non-good	46.7	39.5	0.272	1.312	2.630	0.105
Year 3						
Fail/Non-fail	0	0	N.A.^i^	N.A.	N.A.	N.A.
Good/Non-good	62.3	49.8	0.494	1.639	8.424	0.004**
Professional Behavior	% of SP	% of SN	B	OR	Wald	*p*-value
Year 1						
Fail/Non-fail	0.5	0.4	-0.436	0.647	0.124	0.725
Year 2						
Fail/Non-fail	0.0	0.8	N.A.	N.A.	N.A.	N.A.
Year 3						
Fail/Non-fail	0	0	N.A.	N.A.	N.A.	N.A.
Excellent/Non-excellent	12.1	6.8	0.580	1.785	3.343	0.067
Portfolio						
Year 1						
Fail/Non-fail	1.3	4.0	-1.228	0.293	4.931	0.026*
**Mixed outcomes**						
OSCE	% of SP	% of SN	B	OR	Wald	*p*-value
Year 1						
Fail/Non-fail	8.7	11.2	-0.397	0.673	1.961	0.161
Good/Non-good	38.6	32.0	0.433	1.542	5.653	0.017*
Year 2						
Fail/Non-fail	4.6	5.4	-0.176	0.839	0.218	0.641
Good/Non-good	61.3	51.4	0.407	1.502	5.794	0.016*
Year 3						
Fail/Non-fail	2.0	5.4	-1.023	0.359	4.482	0.034*
Good/Non-good	52.9	38.0	0.608	1.837	12.149	0.000**
**General outcomes**						
Drop-out	% of SP	% of SN	B	OR	Wald	*p*-value
Year 1	3.0	4.5	-0.366	0.694	0.787	0.375
Entire bachelor	3.5	6.2	-0.566	0.568	2.335	0.127
Study delay	% of SP	% of SN	B	OR	Wald	*p*-value
Bachelor	19.2	25.5	-0.359	0.698	3.470	0.062
ECTS	SP; M (SD)	SN; M (SD)	B	SRC	t-value	*p*-value
Year 3, including resits	166.5 (35.5)	161.2 (42.6)	4.689	0.060	1.590	0.112

^a^ M (SD) = Mean (Standard Deviation). ^b^ B = Regression coefficient. ^c^ SRC=Standardized Regression Coefficient. ^d^ OR=Odds Ratio. ^e^ Wald = Wald statistic. ^f^ CAT = Critical Appraisal of a Topic. ^g^ CORE = Consulting and Reflecting skills. ^h^ OSCE = Objective Structured Clinical Examination. ^i^ N.A.= Not Applicable. *p < 0.05, ** *p* < 0.005

## Discussion

Backward chaining from the CanMEDS framework was used to develop an outcome-based selection procedure for medical school. This procedure addressed the whole range of competences, from academic achievement to (inter)personal attributes. We found that the students selected through this procedure significantly outperformed their counterparts who were primarily rejected in the same selection process but were then admitted through an alternative route. Differences in study performance in favor of the selection-positive students were seen across the full range of cognitive, (inter)personal, and mixed outcomes, and throughout the entire three-year bachelor in medicine.

Our finding that selection-positive students performed better than the selection-negative ones on cognitive outcomes was surprising in light of the fact that their pu-GPA did not differ. This indicates incremental validity of our selection procedure over pu-GPA. The significant differences between the selection-positive and selection-negative students persisted throughout the three-year bachelor. Earlier studies showed that the predictive value of pu-GPA for academic achievement decreases after the first year of medical school [1, 2]. The persisting predictive value is consistent with literature on aptitude tests (e.g. [26, 34]), and therefore likely due to selection.

There were only few fails in the end-of-year summative assessments of (inter)personal skills (0–2.4% per outcome measure) and their discriminative value was low. Nevertheless, selection-positive students performed significantly better than selection-negative students, especially with respect to their communication and reflection skills and their portfolio. While almost all students passed the assessment of their professional behavior, selected students were more likely to receive *Excellent* scores at the end of their bachelor. These findings are in line with previous research on the predictive value of SJTs for (inter)personal performance [25, 35], stating that the predictive value persists over a number of years and predicts performance beyond the cognitively-based pu-GPA.

Interestingly, our combination of tools seems (increasingly) proficient in predicting OSCE performance. So far, OSCE performance has mostly been predicted by MMIs [1, 36], with emerging evidence that SJTs may also be predictive [35]. Aptitude tests, on the other hand, do not appear to predict clinical or pre-clinical OSCE performance [37]. The observed predictive value for the OSCEs in our study inspires confidence with respect to the performance of selected students in the master-phase, where they have to perform in a clinical environment.

General outcomes did not show significant differences between selection-positive and selection-negative students, possibly because of the low frequency of drop-out. Interestingly, other studies from the Netherlands have identified that taking part in a selection process significantly reduces drop-out [22, 38]. This is consistent with our situation; students who entered medical school by lottery only (without participating in the selection procedure) were more likely (about 2.5–2.9 times) to drop-out than selected students [39].

One of the strengths of this study is that the selection procedure somewhat resembled programmatic assessment [40]: combining a number of selection tools with evidence-base [1, 2] as well as the judgments of a variety of examiners (SMEs) to obtain a holistic impression of the applicants. The rater-reliability and internal reliability of the V-SJT and aptitude tests proved acceptable, especially considering the fact that they combined the assessment of multiple competences. These findings are in line with reviews in this field that have shown good psychometric qualities for SJTs and well-designed aptitude tests [1, 2, 41]. Furthermore, applicants in all cohorts agreed that the selection procedure assessed characteristics of importance for a medical career (supporting face validity). Another strength of this study is the inclusion of three student cohorts that were followed longitudinally throughout their entire three-year bachelor of medicine. This kind of longitudinal research investigating selection procedures as a whole has been rare, and there have been calls for more of these studies [2, 7]. In addition, the selection-positive students could be compared to selection-negative students within the same cohort, namely the students who were rejected in the same selection procedure but entered medical school through the national weighted lottery.

There are several limitations in the current study that should be kept in mind. Firstly, this was a single-site study, and generalizations to other contexts should be done with caution. However, the use of an internationally known and well-established outcome framework benefits generalizability. It is important to note that the current selection procedure was implemented in a context in which medical schools are considered to be of equal quality. This differs from the situation in other countries, such as the USA and UK, where medical schools are ranked. Secondly, the current study reports on results from the pre-clinical bachelor-phase alone; future research should examine differences across groups in the clinical phase of medical school. Related to the selection procedure itself, there is no way to guarantee that applicants fill in the first-round portfolio themselves. They could receive help from others, or others could even write it for them. However, with the evidence-burden built into this portfolio, this should not affect the applicants’ chances of getting into round two. Furthermore, the applicants’ score in round one is not taken into account once round two is reached. Lastly, the absence of a face-to-face element in the selection procedure could be seen as a weakness of the selection procedure. On the other hand, including a face-to-face element may also introduce bias [1, 2, 42]. In addition, the chosen approach to selection, having the applicants fill out an online portfolio at home, was found to enable feasible, robust pre-screening at a distance for large numbers of applicants.

## Conclusions

All in all, we have shown that an outcome-based, holistic selection procedure is predictive of study success across a variety of cognitive, (inter)personal skills and mixed assessments. Although we did not carry out direct comparisons with other tools, our outcome-based approach seems to address some of the limitations of individual selection tools in relation to predictive validity [7, 10, 13, 15, 43]. We urge others to consider designing and implementing outcome-based selection aligned with curricula and assessment processes, and encourage robust evaluations of the predictive validity of this approach in other contexts, as well as throughout the clinical years and beyond.

## Abbreviations

(pu-)GPA: (pre-university) Grade Point Average
(V-)SJT: (Video-based) Situational Judgment Test
CanMEDS: Canadian Medical Education Directives for Specialists
CASPer: Computer-based Assessment for Sampling Personal characteristics
CAT: Critical Appraisal of a Topic
CORE: Consulting and Reflecting skills
ECTS: European Credit Transfer System
MMI: Multiple Mini Interviews
MUMS: Maastricht University Medical School
NVMO: Nederlandse Vereniging voor Medisch Onderzoek; Netherlands Association for Medical Education
OR: Odds Ratio
OSCE: Objective Structured Clinical Examination
PBL: Problem-Based Learning
SME: Subject Matter Expert
SN: Selection-negative students; the group of students that was rejected in the university-based selection but got into medical school through a national lottery procedure
SP: Selection-positive; the group of students that was selected
SRC: Standardized Regression Coefficient

## References

[1] Cleland J, Dowell J, McLachlan J, Nicholson S, Patterson F. Identifying best practice in the selection of medical students (literature review and interview survey); 2012. https://www.sgptg.org/app/download/7964849/Identifying_best_practice_in_the_selection_of_medical_students.pdf_51119804.pdf. Accessed 02 July 2015.

[2] Patterson F, Knight A, Dowell J, Nicholson S, Cousans F, Cleland J (2016). How effective are selection methods in medical education? A systematic review. Med Educ.

[3] Prideaux D, Roberts C, Eva K, Centeno A, Mccrorie P, Mcmanus C, Patterson F, Powis D, Tekian A, Wilkinson D (2011). Assessment for selection for the health care professions and specialty training: consensus statement and recommendations from the Ottawa 2010 conference. Med Teach.

[4] Girotti JA, Park YS, Tekian A (2015). Ensuring a fair and equitable selection of students to serve society's health care needs. Med Educ.

[5] Patterson F, Lievens F, Kerrin M, Munro N, Irish B (2013). The predictive validity of selection for entry into postgraduate training in general practice: evidence from three longitudinal studies. Brit J Gen Pract.

[6] Burns CA, Lambros MA, Atkinson HH, Russell G, Fitch MT (2017). Preclinical medical student observations associated with later professionalism concerns. Med Teach.

[7] Patterson F, Cleland J, Cousans F (2017). Selection methods in healthcare professions: where are we now and where next?. Adv Health Sci Educ.

[8] Dore KL, Roberts C, Wright S (2017). Widening perspectives: reframing the way we research selection. Adv Health Sci Educ.

[9] MacKenzie RK, Dowell J, Ayansina D, Cleland JA (2016). Do personality traits assessed on medical school admission predict exit performance? A UK-wide longitudinal cohort study. Adv Health Sci Educ.

[10] Powis D (2015). Selecting medical students: an unresolved challenge. Med Teach.

[11] Bandiera G, Maniate J, Hanson MD, Woods N, Hodges B (2015). Access and selection: Canadian perspectives on who will be good doctors and how to identify them. Acad Med.

[12] Hautz SC, Hautz WE, Feufel MA, Spies CD (2015). Comparability of outcome frameworks in medical education: implications for framework development. Med Teach.

[13] Hecker K, Norman G (2017). Have admissions committees considered all the evidence?. Adv Health Sci Educ.

[14] Cleland J, Dowell J, Nicholson S, Patterson F: How can greater consistency in selection between medical schools be encouraged? A project commissioned by the Selecting for Excellence Group (SEEG). 2014. https://www.medschools.ac.uk/media/2447/selecting-for-excellence-research-professor-jen-cleland-et-al.pdf. Accessed 21 Feb 2017.

[15] Wilkinson TM, Wilkinson TJ (2016). Selection into medical school: from tools to domains. BMC Med Educ.

[16] Siu E, Reiter HI (2009). Overview: What’s worked and what hasn’t as a guide towards predictive admissions tool development. Adv Health Sci Educ.

[17] Frank JR, Snell L, Englander R, Holmboe ES (2017). Implementing competency-based medical education: moving forward. Med Teach.

[18] Holmboe ES, Edgar L, Hamstra S. In: ACGME, editor. The Milestones Guidebook. Chicago; 2016. https://www.acgme.org/Portals/0/MilestonesGuidebook.pdf. Accessed 20 Dec 2016.

[19] van Herwaarden CLA, Laan RFJM, Leunissen RRM. Raamplan Artsopleiding 2009. Utrecht: Nederlandse Federatie van Universitair Medische Centra; 2009.

[20] Frank JR: The CanMEDS 2005 physician competency framework: Better standards, better physicians, better care. Royal College of Physicians and Surgeons of Canada; 2005. http://www.ub.edu/medicina_unitateducaciomedica/documentos/CanMeds.pdf. Accessed 25 Aug 2015.

[21] Holmboe ES, Sherbino J, Englander R, Snell L, Frank JR (2017). A call to action: the controversy of and rationale for competency-based medical education. Med Teach.

[22] Schripsema NR, van Trigt AM, Borleffs JCC, Cohen-Schotanus J (2014). Selection and study performance: comparing three admission processes within one medical school. Med Educ.

[23] Frank JR, Snell LS, Sherbino J (2014). The draft CanMEDS 2015 physician competency framework - series ii.

[24] Dore KL, Reiter HI, Eva KW, Krueger S, Scriven E, Siu E, Hilsden S, Thomas J, Norman GR (2009). Extending the interview to all medical school candidates -- computer-based multiple sample evaluation of noncognitive skills (CMSENS). Acad Med.

[25] Dore KL, Reiter HI, Kreuger S, Norman GR (2017). CASPer, an online pre-interview screen for personal/professional characteristics: prediction of national licensure scores. Adv Health Sci Educ.

[26] Emery JL, Bell JF (2009). The predictive validity of the BioMedical admissions test for pre-clinical examination performance. Med Educ.

[27] Patterson F, Zibarras L, Ashworth V (2015). Situational judgement tests in medical education and training: research, theory and practice: Amee guide no. 100. Med Teach.

[28] de Brouwer CPM, Kant I, Smits LJM, Voogd AC. Training critical appraisal of a topic. Een onmisbare handleiding in het tijdperk van Evidence Based Medicine. Maastricht: Mediview; 2009.

[29] Tio RA, Schutte B, Meiboom AA, Greidanus J, Dubois EA, Bremers AJA (2016). The Dutch working Group of the Interuniversity Progress Test of M: the progress test of medicine: the Dutch experience. Perspect Med Educ.

[30] Lehmann EL, D'Abrera HJM (1975). Nonparametrics: statistical methods based on ranks.

[31] Field A (2009). Discovering statistics using SPSS (and sex, drugs and rock 'n' roll).

[32] Rosenthal JA (1996). Qualitative descriptors of strength of association and effect size. J Soc Serv Res.

[33] Cohen J. Statistical power analysis for the behavioral sciences. 2nd ed. London: Routledge; 1988.

[34] de Visser M, Fluit C, Fransen J, Latijnhouwers M, Cohen-Schotanus J, Laan R (2016). The effect of curriculum sample selection for medical school. Adv Health Sci Educ.

[35] Lievens F (2013). Adjusting medical school admission: assessing interpersonal skills using situational judgement tests. Med Educ.

[36] Kelly M, Dowell J, Husbands A, Newell J, O'Flynn S, Kropmans T, Dunne F, Murphy A (2014). The fairness, predictive validity and acceptability of multiple mini interview in an internationally diverse student population- a mixed methods study. BMC Med Educ.

[37] Husbands A, Mathieson A, Dowell J, Cleland J, MacKenzie R (2014). Predictive validity of the UK clinical aptitude test in the final years of medical school: a prospective cohort study. BMC Med Educ.

[38] Wouters A, Croiset G, Galindo-Garre F, Kusurkar RA (2016). Motivation of medical students: selection by motivation or motivation by selection. BMC Med Educ.

[39] Schreurs S, Cleland J, Muijtjens AMM, oude Egbrink MG, Cleutjens K. Does selection pay off? A cost–benefit comparison of medical school selection and lottery systems. Med Educ. 10.1111/medu.13698. 10.1111/medu.13698 PMC6282742 30324680

[40] van der Vleuten CPM, Schuwirth LWT (2005). Assessing professional competence: from methods to programmes. Med Educ.

[41] De Leng WE, Stegers-Jager KM, Husbands A, Dowell JS, Born MP, Themmen APN (2017). Scoring method of a situational judgment test: influence on internal consistency reliability, adverse impact and correlation with personality?. Adv Health Sci Educ.

[42] Griffin BN, Wilson IG (2010). Interviewer bias in medical student selection. MJA.

[43] Frenk J, Chen L, Bhutta ZA, Cohen J, Crisp N, Evans T, Fineberg H, Garcia P, Ke Y, Kelley P (2010). Health professionals for a new century: transforming education to strengthen health systems in an interdependent world. Lancet.

